# Insights into the historical trajectory and research trends of immune checkpoint blockade in colorectal cancer: visualization and bibliometric analysis

**DOI:** 10.3389/fimmu.2024.1478773

**Published:** 2024-10-31

**Authors:** Yonglong Chang, Xuhui Zhou, Kechao Nie, Jinhui Liu, Sifang Zhang

**Affiliations:** ^1^ Department of Integrated Traditional Chinese & Western Medicine, The Second Xiangya Hospital, Central South University, Changsha, Hunan, China; ^2^ National Clinical Research Center for Metabolic Diseases The Second Xiangya Hospital, Central South University Changsha China, Changsha, Hunan, China; ^3^ Department of Addiction Medicine, Hunan Institute of Mental Health, Brain Hospital of Hunan Province (The Second People’s Hospital of Hunan Province, Changsha, Hunan, China; ^4^ College of Integrated Traditional Chinese & Western Medicine, Hunan University of Traditional Chinese Medicine, Changsha, Hunan, China

**Keywords:** colorectal cancer, immune checkpoint blockade, bibliometric analysis, R-bibliometrix, research trends

## Abstract

**Background:**

Colorectal cancer (CRC) is a malignant tumor that poses a significant threat to human health due to rising incidence and mortality rates. In recent years, immune checkpoint blockade (ICB) therapy, represented by Programmed death receptor 1 (PD-1), T-lymphocyte-associated protein 4 (CTLA-4), and others, has been widely applied in CRC and has achieved encouraging results in some patients and has become a hot topic in both clinical and basic research.

**Objective:**

This study undertakes a comprehensive bibliometric analysis of ICB research in CRC, aiming to evaluate the current status, identify future trends, and provide scientific insights for researchers and decision-makers.

**Methods:**

Utilizing the Web of Science Core Collection (WoSCC), articles focusing on ICB in CRC from 2000 to 2022 were retrieved. Knowledge mapping and bibliometric analysis were conducted using tools such as CiteSpace, VOSviewer, SCImago Graphicay, and the R package bibliometrix.

**Results:**

6,718 publications were analyzed from 24,846 institutions across 639 regions. Temporally, ICB research in CRC is rapidly advancing, led by the USA and China with extensive global collaborations. Sun Yat-sen University from China stands out as the institution with the highest number of publications. Professor Thierry Andre from Sorbonne University in France is identified as a prolific author in this field, engaging in extensive collaboration for clinical trials on a global scale. Publications related to this research topic were published in 1,142 academic journals, demonstrating a positive co-citation relationship. Key clustering and burst terms analysis indicate that current research on ICB in CRC has shifted from basic experiments to clinical trials and from universal healthcare to precision medicine.

**Conclusion:**

ICB therapies have shown substantial progress in CRC, highlighting their therapeutic potential. Research trends emphasize deeper drug mechanisms, treatment efficacy prediction, managing immune-related adverse events, and exploring novel drug delivery methods. Collaboration across borders remains crucial for further advancements.

## Introduction

Colorectal cancer (CRC), including colon cancer and rectal cancer, has become the third most common cancer around the world (1). Although the incidence of CRC has declined over the past decade, the mortality rate remains high. According to the International Agency for Research on Cancer, the number of deaths due to CRC in 2020 alone reached 94 million. It is one of the most important challenges to global health (2). While surgery remains the foundation of CRC treatment, patients with recurrent and metastatic disease should receive chemotherapy. Despite new advances in approaches including targeted therapies, the prognosis for advanced CRC remains poor (3). A growing body of evidence suggests that the most promising therapeutic approach is to block immune checkpoint molecules to activate anti-tumor immunity (4). Immune checkpoint blockade (ICB) has revolutionized modern cancer treatment, including CRC, and has attracted significant interest from the oncology community (5). Currently, ICB is widely used in patients with microsatellite instability-high (MSI-H) or mismatch repair deficient (dMMR). Clinical trials of CRC immunotherapy cover advanced backline, first- or second-line, and even early neoadjuvant or adjuvant therapy (6). ICB has taken place in the comprehensive treatment of CRC (7). Examples include T-lymphocyte-associated protein 4 (CTLA-4), programmed death receptor 1 (PD-1), and PD-1 (PD-L1). Given the relatively low incidence of MSI-H: dMMR in advanced or metastatic CRC (3%-5%), understanding this limited research is crucial to support the integrity of CRC immunotherapy research, which may change the clinical practice of the application of ICBs to CRC (8). Due to the biological and clinical characteristics of CRC, the treatment of CRC with ICB has been extensively and intensively studied over the past two decades and has gradually become a research hotspot (9). The analysis of the existing literature reports can help to reveal the research trends in the application of ICBs to CRC and help to provide new insights for future research on CRC immunotherapy.

Bibliometrics, the analysis of published information and its associated data to understand the current state and trends of research across the field, has become an important tool for exploring research areas (10). In the health sector, bibliometrics is often used to measure the impact of articles, assess the impact of selected research articles on future research, and gain insights into trends in the field of research, the results of which are important for both researchers and funders (11). However, to the best of our knowledge, there is no bibliometric analysis of ICB for CRC. In this study, we performed a comprehensive bibliometric analysis over recent decades to help identify research hotspots, predict trends, and fill existing knowledge gaps in the field.

This study visualizes the distribution of annual publications, countries, institutions, authors, source journals, keyword co-occurrences, and co-citations of Web of Science Core Composite (WoSCC) from 2000 to 2022. In addition, we systematically analyze keyword bursts to identify emerging trends and research hotspots, offering new perspectives for ICB research in the field of CRC treatment. By highlighting these trends, we aim to guide future research directions that could address current challenges and barriers in the field.

## Methods

### Data source and search strategy

Bibliographic data are from the Web of Science Core Collection (WoSCC). These include Science Citation Index Expanded, Social Sciences Citation Index, Arts & Humanities Citation Index, Emerging Sources Citation Index, Current Chemical Reactions and Index Chemicus (12). To avoid bias caused by the daily database updates, all the publications from 2000 to 2022 were retrieved and downloaded on November 16, 2023. The search strategy was as follow: (TS=(ipilimumab OR pembrolizumab OR nivolumab OR immunotherapy OR “immune checkpoint blockade” OR “immune checkpoint inhibitor” OR PD-1 OR PD-L1 OR CTLA-4) OR TI=(ipilimumab OR pembrolizumab OR nivolumab OR immunotherapy OR “immune checkpoint blockade” OR “immune checkpoint inhibitor” OR PD-1 OR PD-L1 OR CTLA-4 OR yervoy OR Keytruda OR opdivo) OR AB=(ipilimumab OR pembrolizumab OR nivolumab OR immunotherapy OR “immune checkpoint blockade” OR “immune checkpoint inhibitor” OR PD-1 OR PD-L1 OR CTLA-4 OR yervoy OR Keytruda OR opdivo)) AND (TS=(“Rectal Neoplasm” OR “Rectal Tumor” OR “Rectal Cancer” OR “Rectum Neoplasm” OR “Rectum Cancer” OR “Cancer of the Rectum” OR “Cancer of Rectum” OR “Colorectal Neoplasm” OR “Colorectal Tumor” OR “Colorectal Cancer” OR “Colorectal Carcinoma” OR “Colonic Neoplasm” OR “Colon Neoplasm” OR “ Cancer of Colon” OR “Colon Cancer” OR “Cancer of the Colon” OR “Colonic Cancer” OR “CRC”) OR TI=(“Rectal Neoplasm” OR “Rectal Tumor” OR “Rectal Cancer” OR “Rectum Neoplasm” OR “Rectum Cancer” OR “Cancer of the Rectum” OR “Cancer of Rectum” OR “Colorectal Neoplasm” OR “Colorectal Tumor” OR “Colorectal Cancer” OR “Colorectal Carcinoma” OR “Colonic Neoplasm” OR “Colon Neoplasm” OR “ Cancer of Colon” OR “Colon Cancer” OR “Cancer of the Colon” OR “Colonic Cancer” OR “CRC”) OR AB=(“Rectal Neoplasm” OR “Rectal Tumor” OR “Rectal Cancer” OR “Rectum Neoplasm” OR “Rectum Cancer” OR “Cancer of the Rectum” OR “Cancer of Rectum” OR “Colorectal Neoplasm” OR “Colorectal Tumor” OR “Colorectal Cancer” OR “Colorectal Carcinoma” OR “Colonic Neoplasm” OR “Colon Neoplasm” OR “ Cancer of Colon” OR “Colon Cancer” OR “Cancer of the Colon” OR “Colonic Cancer” OR “CRC”)). The detailed search strategy is shown in **Supplementary Material 1** (13–15). Finally, only the research papers and review articles in English were considered for this study.

**Figure 1:**
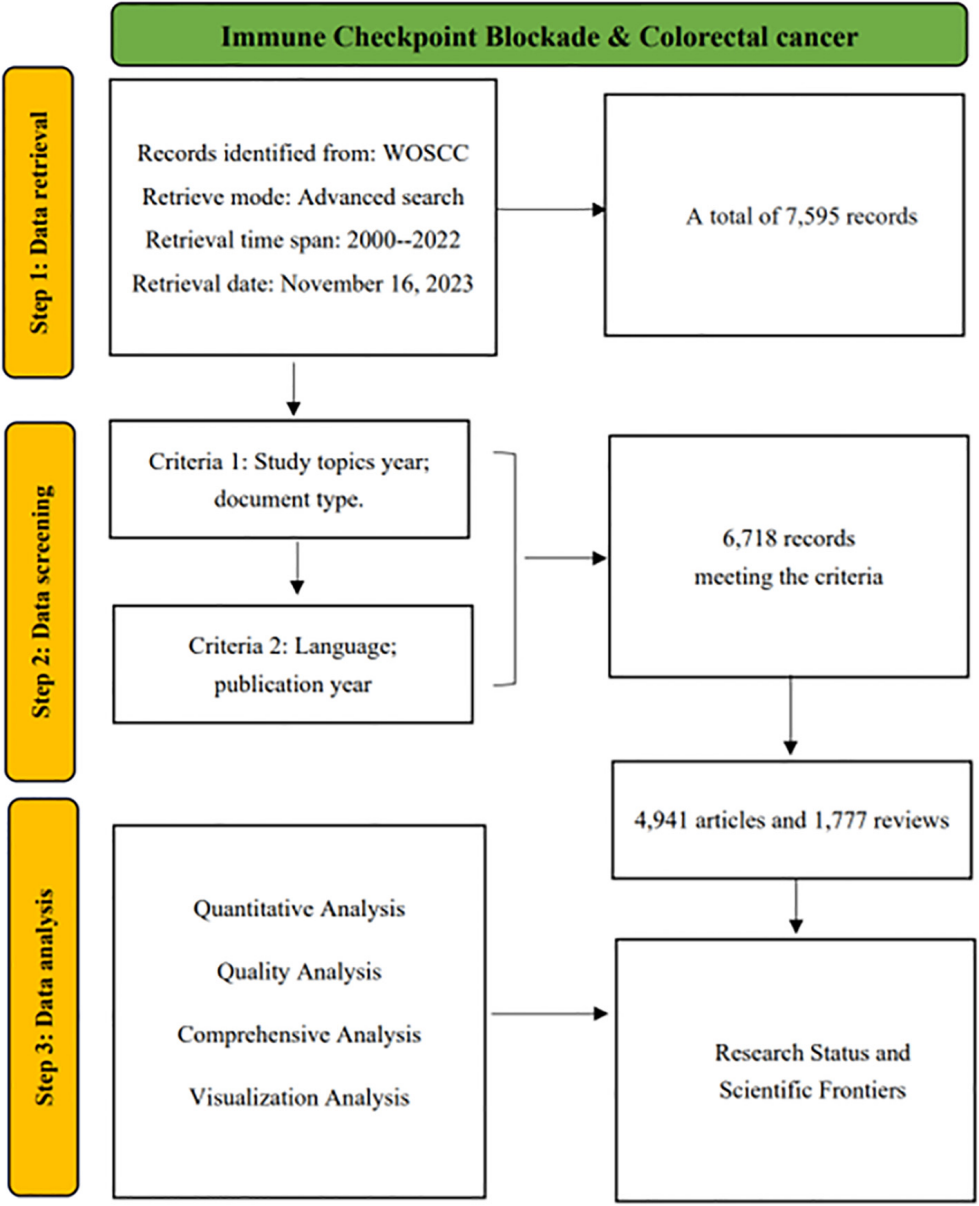
Data screening and research flowchart.

A total of 7,595 documents were retrieved from WoSCC, and after excluding non-research papers and review articles there were 6,786 that met the criteria, and after limiting the language to English, there were still 6,718 publications met the criteria for bibliometric analysis and visualization. The detailed flowchart of the publication screening process is shown in **Figure 1**. This study aligns with the Consolidated Criteria for Reporting Qualitative Research (COREQ) (16).

### Data analysis

The visualization and bibliometric analysis of CRC ICB-related publications includes publication trends, country/region, institution/organization, including journals, authors, keywords, and distribution of major references. Based on the visualization we further performed a comprehensive analysis. In this study, CiteSpace (version 6.1) (17), VOSviewer (version 1.6.18) (18), SCImago Graphica (version 1.0.36), and the R (version 4.1) software package “bibliometrix” (https://www.bibliometrix.org) were used to perform the data analysis and visualization. Moreover, Microsoft Excel 2010 was used to quantitatively visualize the data. Specifically, the bibliometrix package was used to extract and visualize data on the distribution of the top 20 core authors and journals in the H-index. CiteSpace was used to extract and visualize the distribution of country/region, institution/organization, and author postings, as well as to include keyword citation burst analysis and co-citation network analysis. In addition, CiteSpace is used to calculate a node’s centrality, which quantifies the significance of a node’s position within a co-occurrence network. The most commonly used metric is betweenness centrality, and in this study, all references to centrality pertain specifically to betweenness centrality (19). This metric measures the percentage of shortest paths in the network that pass through a given node, helping to identify those nodes that play a critical role in maintaining the overall structure, facilitating information flow, and preserving connectivity (20). In this study, a high betweenness centrality value for a node generally indicates its significant influence within the research domain. SCImago Graphicay was used for country/region and author collaboration network mapping, and the rest of the co-occurrence and visualization analyses were performed using VOSviewer. With the use of these tools, we were able to extract key information from numerous publications and generate visual maps that provided valuable insights into our research (13, 21).

## Results

### Annual publication trends and average citations

**Figure 2:**
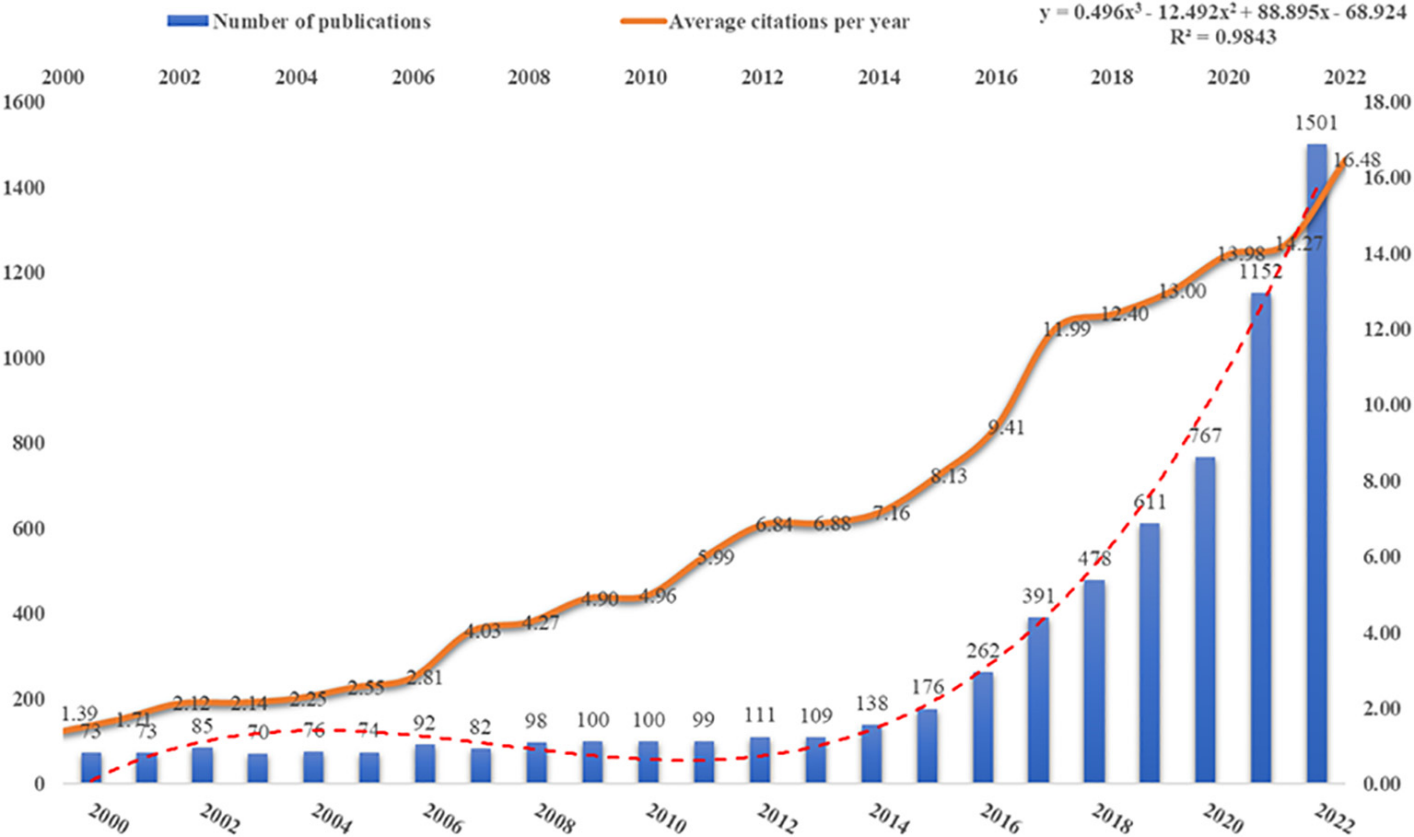
Publications and average annual citations trends.

After inclusion and exclusion criteria, WoS obtained a total of 6,718 articles on ICB in CRC studies. **Figure 2** illustrates the trend of publication numbers versus the average yearly citations from 2000 to 2022. The research on ICB in CRC has been growing rapidly. Especially over the last 5 years, the number of publications accounted for 67.12% of the total. The annual number of publications globally increased from 73 in 2000 to 1,501 in 2022. Before 2019, annual publications were below 500. However, post-2019, they significantly increased, maintaining a steady rise. The average number of citations per year for publications has increased each year and has been above 10 from 2017 to the present. Overall, these findings suggest that ICB research in CRC is experiencing rapid advancement, with increasing interest among researchers in exploring its application in CRC treatment.

### Analysis of distribution and collaboration networks

#### Countries/regions

Regarding geographical distribution, 6,718 publications from 2,846 research institutions across 639 countries/regions were included in the analysis. We calculated the number of publications for each country/region separately and visualized the country/region publication volume and partnerships.

**Figure 3:**
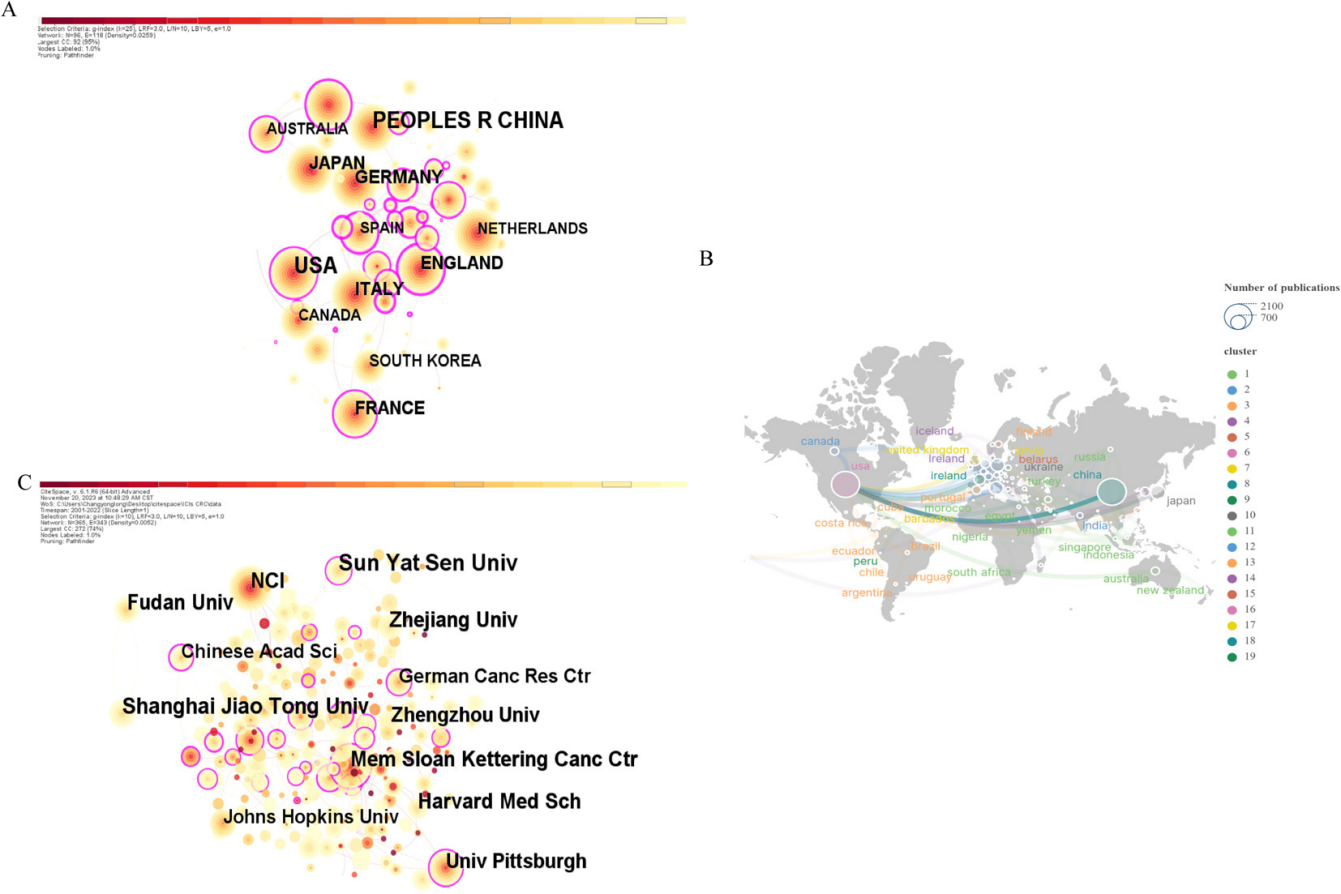
**(A)** CiteSpace-generated national/regional co-occurrence network map. **(B)** Cooperation network map between countries/regions created by SCImago Graphics. **(C)** Research institution co-occurrence network map generated by CiteSpace.

**Figure 3A** displays a co-occurrence map of country/region contributions, where the circle size denotes the number of contributions. **Table 1** presents the top 10 most productive countries/regions. Notably, China leads in publication count (2,002/6,718 publications, 29.8%), followed by the USA (1,898/6,718 publications, 28.3%). However, despite China’s dominance in publication volume, its betweenness centrality value is significantly lower compared to countries like the USA, the UK, and Spain. This lower centrality suggests that while China has a high number of publications, its role as a bridge between different research communities is limited, thus indicating a lower global impact or influence in shaping cross-national research trends.

**Table 1:** Top 10 most productive countries/regions.

Rank	Country	Records	Centrality
1	China	2002	0
2	USA	1898	0.16
3	Japan	515	0
4	Italy	468	0
5	Germany	447	0
6	England	328	0.54
7	France	307	0.12
8	Korea	229	0.08
9	Spain	199	0.63
10	Netherlands	195	0.04

Regarding cooperative relationships, **Figure 3B** highlights the USA’s extensive collaborations worldwide, underlining its significant role in this domain. Nodes with high betweenness centrality, such as the USA, are key in facilitating international collaboration, connecting various research clusters, and driving global research trends. It’s noteworthy that these collaborations are primarily with developed European countries, implying a potential link between a country’s economic development and its scientific research level.

#### Institutions

Generally, the current research of ICB in CRC is conducted in about 2,4846 institutions worldwide. The co-occurrence network of institutional publications is shown in **Figure 3C**. The top 10 institutions in terms of publication output and their centrality are shown in **Table 2**, with Sun Yat-sen University from China ranking first in terms of output (n=155), followed by Shanghai Jiao Tong University, also from China (n=123). China holds five spots among the top 10 institutions in terms of publications. However, despite this high publication volume, the centrality values of Chinese institutions are relatively low compared to their US counterparts, indicating that while Chinese institutions contribute significantly to the field, their role as key connectors or influencers in global research networks is limited.

**Table 2:** Top 10 most productive Institutions.

Rank	Institution	Records	Centrality
1	Sun Yat-Sen University	155	0.1
2	Shanghai Jiao Tong University	123	0.03
3	Memorial Sloan Kettering Cancer Canter	118	0.35
4	The University of Texas MD Anderson Cancer Canter	116	0.05
5	Fudan University	113	0.05
6	Harvard Medical School	106	0
7	NCI	101	0.05
8	Zhejiang University	92	0.01
9	Zhengzhou University	75	0.01
10	University of Pittsburgh	75	0.2

Moreover, Memorial Sloan Kettering Cancer Center in the USA tops in centrality with 0.35, highlighting its crucial role as a bridge between different research institutions and its influence in shaping global collaboration trends. This suggests that while Chinese institutions are active in advancing this research area, institutions from the USA are more central to global collaborations and research dissemination.

#### Authors

**Figure 4:**
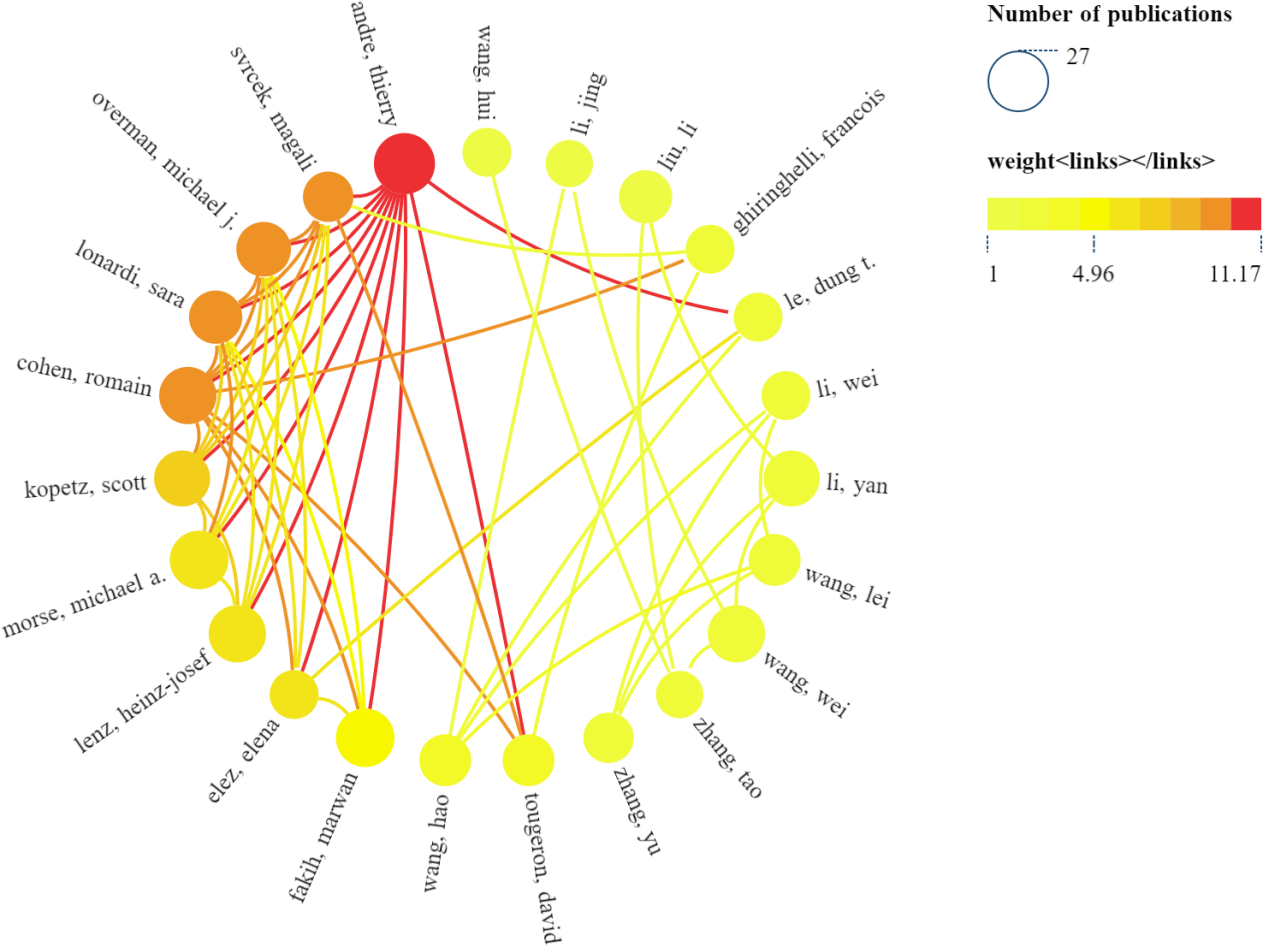
Top 30 authors’ collaboration network map produced by SCImago Graphicay.

Numerous scholars have devoted themselves to exploring the role of ICB therapy in CRC. The publications included in this study involved a total of 4,0814 researchers. **Table 3** provides details about the top 10 authors in terms of publications, all of whom have published significant articles in this field. Professor Thierry Andre from Sorbonne University, France, leads the list. He has long been interested in immunotherapy for CRC and cancers with microsatellite instability/DNA mismatch repair defects. At the same time, we used SCImago Graphicay to map the collaboration network of the top 30 authors in terms of publications, as shown in **Figure 4**, and found that Thierry Andre has established collaborations with the most prolific authors around the world. Professor Heinz-Josef Lenz from the University of Southern California, Norris, USA, ranked second. He was the first to detect intratumoral RNA levels linked to 5-FU and oxaliplatin efficacy and led the first prospective randomized phase II trial using FFPE samples for gene expression. Moreover, he identified primary tumor location in CRC as an independent predictive and prognostic marker, now incorporated in the National Comprehensive Cancer Network (NCCN) guidelines. Four of the top 10 authors by publications hail from the USA, and their exceptional contributions have significantly contributed to the USA’s leadership role in this research domain. Notably, all top 10 authors are from developed nations and are committed to investigating the clinical applications of CRC immunotherapy.

**Table 3:** Top 10 most productive authors.

Rank	Author	Records	Country	Centrality
1	Thierry Andre	26	France	0.02
2	Heinz-Josef Lenz	19	USA	0.01
3	Romain Cohen	19	France	0.02
4	Chiara Cremolini	18	Italy	0.01
5	Filippo Pietrantonio	18	Italy	0.02
6	Scott Kopetz	17	USA	0
7	Magali Svrcek	15	France	0
8	Michael J Overman	14	USA	0
9	Sara Lonardi	14	Italy	0
10	Marwan Fakih	14	USA	0

#### Journals and co-cited journals

**Figure 5:**
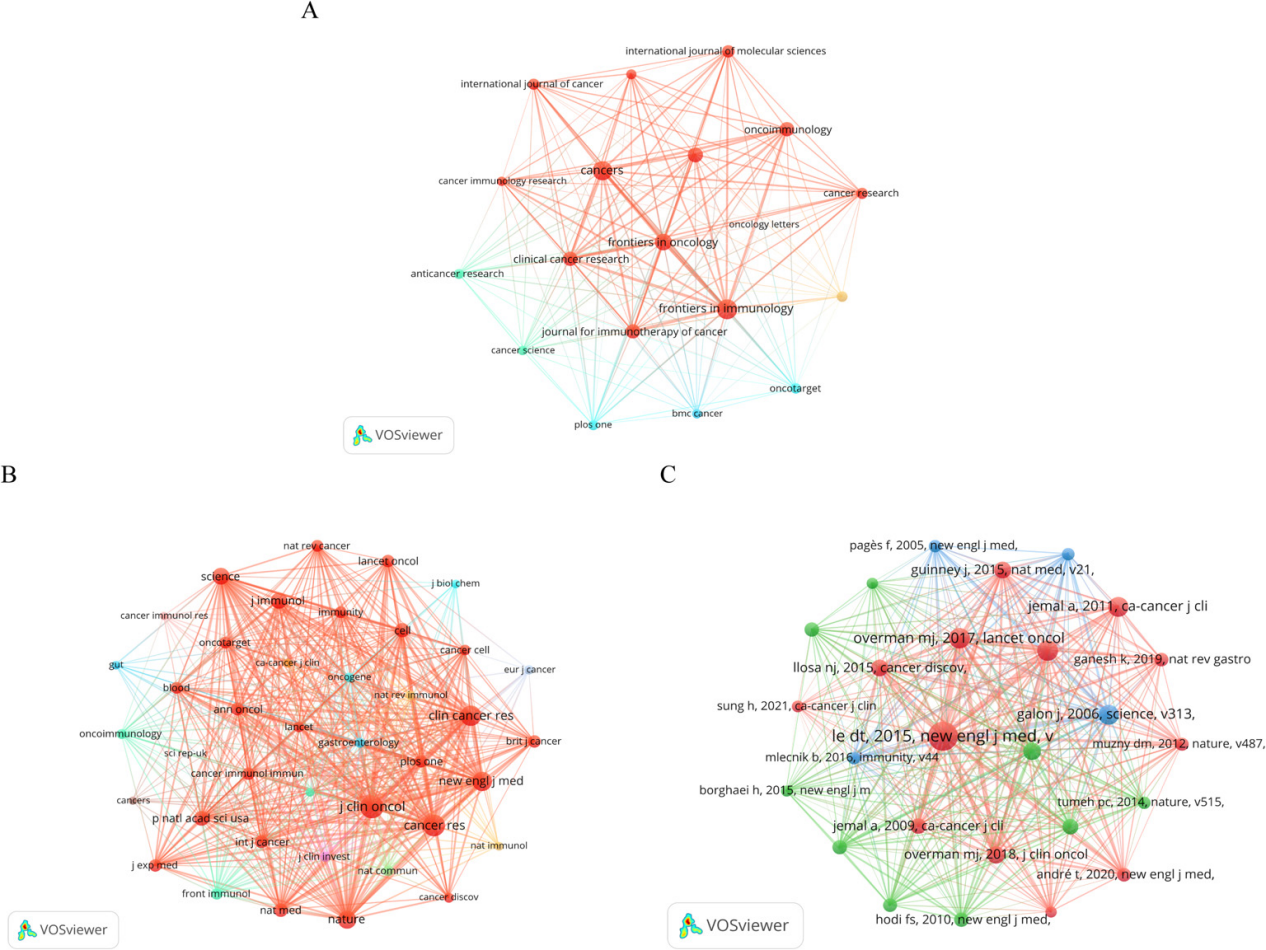
**(A)** Journal co-occurrence network map created by VOSviewer. The threshold is set to a minimum number of documents from the source of 50. **(B)** Journal co-citation network map created by VOSviewer. The threshold is set at a minimum of 2,000 citations from sources. **(C)** Network map of co-cited documents created by VOSviewer. The threshold is set at a minimum of 200 citations for cited references.

**Table 4** presents the top 10 journals based on publication volume and the 10 journals with the highest number of citations in this research domain. “*Frontiers in Immunology*” leads with 253 publications, followed by “*Cancers*” (n=239) and “*Frontiers in Oncology*” (n=175). This dominance of highly reputable journals underscores the growing interest and high impact of ICB research in CRC. Remarkably, 90% were categorized as Q1 in Journal Citation Reports (JCR), highlighting the high research significance of this area. We further screened journals with at least 50 publications in this field and mapped the journal co-occurrence network. **Figure 5A** illustrates 19 journals grouped into three clusters, with larger nodes indicating higher publication counts. The clustering reflects distinct research focuses within ICB in CRC, with each cluster representing a thematic concentration in the field. Lines between nodes signify cross-citation relationships among the journals. As for the 10 most co-cited journals, all have more than 2,500 citations each. *“Cancer Research”* topped the list with 4,463 citations. It was followed by *“Clinical Cancer Research”* (Co-citation=4402), and *“Journal of Clinical Oncology”* was third with 3,857 citations. These journals serve as key sources of knowledge, frequently referenced in CRC-ICB research, underscoring their foundational role in the field. Ninety percent of the top 10 co-cited journals were in JCR Region I. Seven journals boasted an impact factor exceeding 10, led by “*The New England Journal of Medicine*” (IF=96.2) and “*Nature Medicine*” (IF=58.7). Notably, “*Journal of Immunology*” with an impact factor of 3.6, garnered 2,655 citations, showcasing the significant interest in this research field. This suggests that journals with lower impact factors can still have a strong influence in niche or emerging fields. Similarly, we created a network of co-cited journals depicted in **Figure 5B**, forming six clusters with positive co-citation relationships among the journals.

**Table 4:** Top 10 most productive journals and co-cited journals.

Rank	Journal	Records	JCR/IF(2023)	Rank	Co-cited Journal	Records	JCR/IF(2023)
1	Frontiers in Immunology	253	Q1/5.7	1	Cancer Research	4463	Q1/12.5
2	Cancers	239	Q1/4.5	2	Clinical Cancer Research	4402	Q1/10
3	Frontiers in Oncology	175	Q2/3.5	3	Journal of Clinical Oncology	3857	Q1/42.1
4	Cancer Immunology, Immunotherapy	145	Q1/4.6	4	The New England Journal of Medicine	3719	Q1/96.2
5	Clinical Cancer Research	130	Q1/10	5	Nature	3570	Q1/50.5
6	OncoImmunology	124	Q1/6.5	6	Science	3448	Q1/44.7
7	Journal for ImmunoTherapy of Cancer	123	Q1/10.3	7	PNAS	3186	Q1/9.4
8	International Journal of Molecular Sciences	92	Q1/4.9	8	Nature Medicine	3044	Q1/58.7
9	Cancer Research	76	Q1/12.5	9	International Journal of Cancer	2742	Q1/5.7
10	British Journal of Cancer	57	Q1/6.4	10	Journal of Immunology	2655	Q2/3.6

#### Co-cited references and burst references

Co-cited references are those cited in multiple publications within this study. We utilized VoSviewer to visualize the literature’s co-citation network for ICB studies in CRC, depicted in **Figure 5C**. Each node represents a co-cited reference, with larger nodes indicating higher citation frequency. To delve deeper into highly cited literature, we compiled a list of the top 10 cited references, detailed in **Table 5**. The most cited document (n=732) is a 2015 publication from the USA with Prof. Dung T. Le as the first author titled “PD-1 Blockade in Tumors with Mismatch-Repair Deficiency.” The study evaluated pembrolizumab, an anti-PD-1 immune checkpoint inhibitor, in a phase 2 trial involving 41 patients with progressive metastatic cancers, assessing the clinical activity in those with or without mismatch repair deficiency (MMR). The results showed that for CRC patients with mismatch repair defects, the immune-related objective response rate was 40% and the immune-related progression-free survival rate was 78%. For CRC patients with normal mismatch repair, the immune-related objective response rate and immune-related progression-free survival rate were lower. Meanwhile, mismatch repair-deficient non-CRC patients showed similar responses. Overall extra-tissue whole genome sequencing showed that a significantly higher number of somatic mutations in mismatch repair-deficient tumors was associated with longer progression-free survival, and this study confirms that mismatch repair status predicts the clinical benefit of immune checkpoint blockade with pembrolizumab. The second most cited document was a 2017 multicenter, phase 2 clinical trial study by Professor Michael J. Overman, evaluating Nivolumab’s efficacy in treating patients with metastatic CRC, specifically those with defective DNA mismatch repair or high microsatellite instability. This study was published in “*The Lancet Oncology*.” Notably, five of the top 10 co-cited publications were clinical trial studies, suggesting that the current research on ICB in CRC is still emerging and requires further exploration regarding safety and long-term efficacy.

**Table 5:** Top 10 most co-cited publications.

Rank	Co-cited reference	Total Citations	Centrality	Journal	JCR/IF(2023)
1	Le DT, et al. (22)	732	0.25	New England Journal of Medicine	Q1/96.2
2	Overman MJ, et al. (23)	639	0.21	Lancet. Oncology	Q1/41.6
3	Le DT, et al. (24)	624	0.21	Science	Q1/44.7
4	Overman MJ, et al. (8)	481	0.02	Journal of Clinical Oncology	Q1/42.1
5	Llosa NJ, et al. (25)	288	0.02	Cancer Discovery	Q1/29.7
6	André T, et al. (26)	283	0.16	New England Journal of Medicine	Q1/96.2
7	Ganesh K, et al. (27)	280	0.02	Nature Reviews Gastroenterology & Hepatology	Q1/45.9
8	Pagès F, et al. (28)	236	0.02	Lancet	Q1/98.4
9	Routy B, et al. (29)	206	0.11	Science	Q1/44.7
10	Guinney J, et al. (30)	204	0.04	Nature Medicine	Q1/58.7

**Figure 6:**
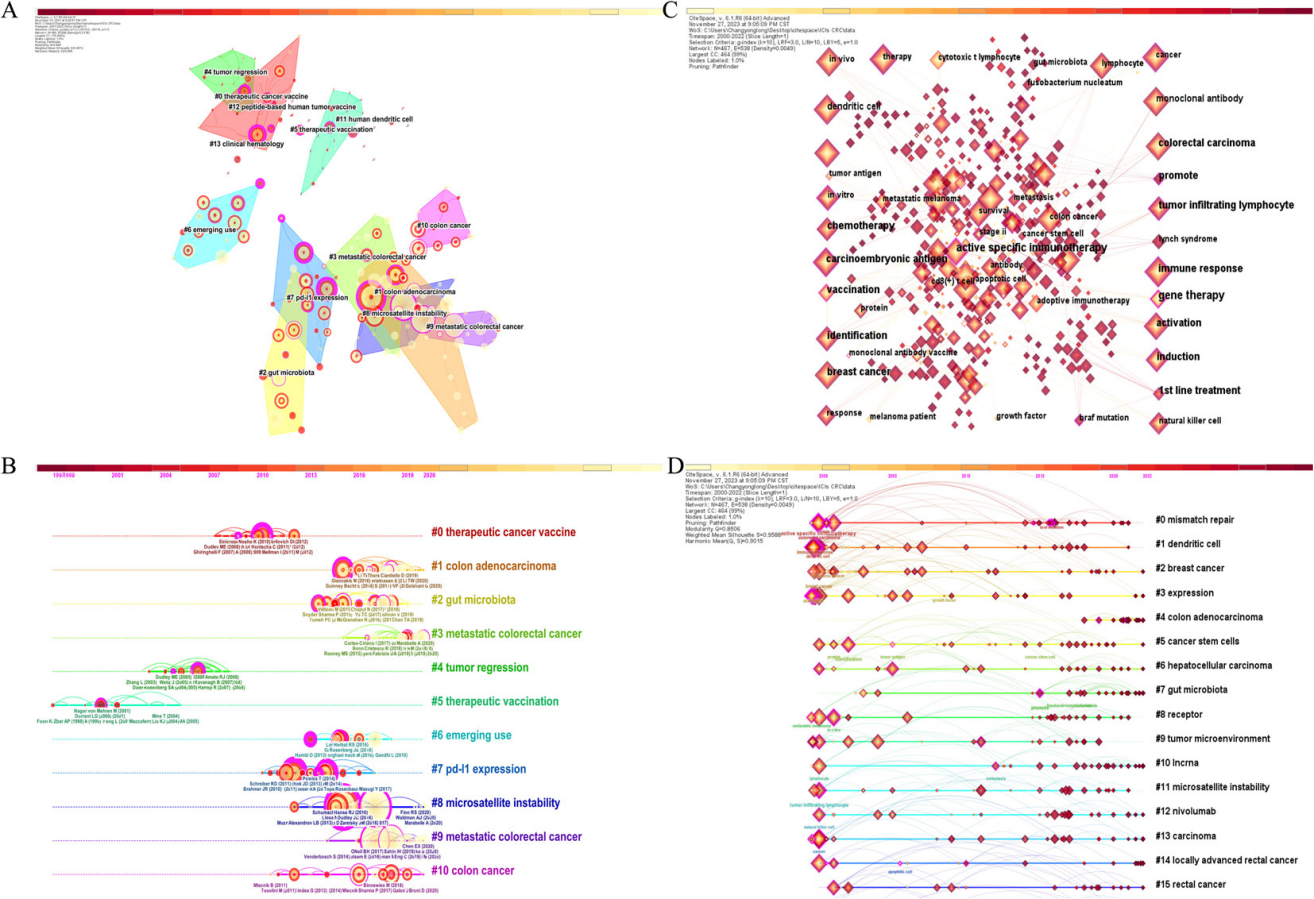
**(A)** Cluster map of co-cited literature created by CiteSpace. **(B)** Timeline cluster map of co-cited literature. **(C)** Keyword co-occurrence network map created by CiteSpace. **(D)** Timeline cluster map of keywords.

These co-cited literature were published in high-quality journals, while the highest impact factor of the published journals was *“Lancet”* (IF=98.4) and the lowest was *“Cancer Discovery”* (IF=29.7). Subsequently, we used the LLR algorithm with the CiteSpace automatic clustering function to cluster the co-cited literature, as shown in **Figure 6A**, where the analysis revealed a modularity Q of 0.845 and a mean silhouette S reaching a high level of 0.9512, indicating a strong clustering effect and a non-homogeneous network. The high modularity and silhouette values suggest that the co-citation network is well-defined and that distinct thematic areas within the research are present. The clusters were numbered in ascending order starting from 0. A total of 14 clusters were formed for co-cited references. To showcase the evolving research hotspots over time, we present the top 10 clusters as a timeline in **Figure 6B**. The largest cluster, labeled #0 therapeutic cancer vaccine, indicates that many studies on CRC and ICB have cited literature from this cluster. The prominence of this cluster highlights the growing interest in therapeutic strategies targeting the immune system in CRC treatment. Cluster labels are numbered in ascending order, with smaller numbers indicating more studies within the corresponding cluster, thus highlighting the importance of therapeutic cancer vaccines in this area of research. In addition, #3 and #9 are both metastatic colorectal cancer, indicating that the current trend of ICB research in CRC may be immunotherapy for metastatic nodal CRC. Scientists are increasingly focusing on the typing of CRC and the corresponding ICB therapy.

Citation bursts in references signify a sudden increase in citations over time, indicating that they are rapidly gaining recognition and dissemination within the research field. **Table 6** displays the top 25 references with the most significant citation bursts. In this table, red lines represent a higher frequency of citations, while blue lines suggest a decline in citations. Particularly noteworthy is the strongest burst of literature (strength=169.29) observed during the period 2016-2020, aligning with the most co-cited literature. This correlation underscores the heightened significance of these studies within the field, suggesting they represent pivotal contributions to advancing knowledge in ICB and CRC. Additionally, we observed that numerous co-cited references on ICB in CRC research from the 1990s to the present remain extensively cited and evolve into the most cited references with strong burst strengths in the years following their publication. This observation indicates that ICB continues to be a prominent research focus in the field of CRC, with ongoing developments that build on foundational studies.

**Table 6:** Top 25 references with the strongest citation bursts.

Rank	References	Year	Strength	Begin	End	2000 - 2022
1	Riethmüller G, 1998, J CLIN ONCOL, V16, P1788, DOI 10.1200/JCO.1998.16.5.1788	1998	36.84	2000	2003	▃▃▃▃▂▂▂▂▂▂▂▂▂▂▂▂▂▂▂▂▂▂▂
2	Vermorken JB, 1999, LANCET, V353, P345, DOI 10.1016/S0140-6736(98)07186-4	1999	35.26	2000	2004	▃▃▃▃▃▂▂▂▂▂▂▂▂▂▂▂▂▂▂▂▂▂▂
3	Galon J, 2006, SCIENCE, V313, P1960, DOI 10.1126/science.1129139	2006	35.25	2007	2011	▂▂▂▂▂▂▂▃▃▃▃▃▂▂▂▂▂▂▂▂▂▂▂
4	Hodi FS, 2010, NEW ENGL J MED, V363, P711, DOI 10.1056/NEJMoa1003466	2010	48.68	2011	2015	▂▂▂▂▂▂▂▂▂▂▂▃▃▃▃▃▂▂▂▂▂▂▂
5	Kantoff PW, 2010, NEW ENGL J MED, V363, P411, DOI 10.1056/NEJMoa1001294	2010	30.45	2011	2015	▂▂▂▂▂▂▂▂▂▂▂▃▃▃▃▃▂▂▂▂▂▂▂
6	Topalian SL, 2012, NEW ENGL J MED, V366, P2443, DOI 10.1056/NEJMoa1200690	2012	89.57	2013	2017	▂▂▂▂▂▂▂▂▂▂▂▂▂▃▃▃▃▃▂▂▂▂▂
7	Brahmer JR, 2012, NEW ENGL J MED, V366, P2455, DOI 10.1056/NEJMoa1200694	2012	61	2013	2017	▂▂▂▂▂▂▂▂▂▂▂▂▂▃▃▃▃▃▂▂▂▂▂
8	Pardoll DM, 2012, NAT REV CANCER, V12, P252, DOI 10.1038/nrc3239	2012	56.57	2014	2017	▂▂▂▂▂▂▂▂▂▂▂▂▂▂▃▃▃▃▂▂▂▂▂
9	Fridman WH, 2012, NAT REV CANCER, V12, P298, DOI 10.1038/nrc3245	2012	35.2	2014	2017	▂▂▂▂▂▂▂▂▂▂▂▂▂▂▃▃▃▃▂▂▂▂▂
10	Wolchok JD, 2013, NEW ENGL J MED, V369, P122, DOI 10.1056/NEJMoa1302369	2013	29.12	2014	2018	▂▂▂▂▂▂▂▂▂▂▂▂▂▂▃▃▃▃▃▂▂▂▂
11	Llosa NJ, 2015, CANCER DISCOV, V5, P43, DOI 10.1158/2159-8290.CD-14-0863	2015	61.15	2015	2020	▂▂▂▂▂▂▂▂▂▂▂▂▂▂▂▃▃▃▃▃▃▂▂
12	Herbst RS, 2014, NATURE, V515, P563, DOI 10.1038/nature14011	2014	42.29	2015	2019	▂▂▂▂▂▂▂▂▂▂▂▂▂▂▂▃▃▃▃▃▂▂▂
13	Tumeh PC, 2014, NATURE, V515, P568, DOI 10.1038/nature13954	2014	40.86	2015	2019	▂▂▂▂▂▂▂▂▂▂▂▂▂▂▂▃▃▃▃▃▂▂▂
14	Snyder A, 2014, NEW ENGL J MED, V371, P2189, DOI 10.1056/NEJMoa1406498	2014	36.21	2015	2019	▂▂▂▂▂▂▂▂▂▂▂▂▂▂▂▃▃▃▃▃▂▂▂
15	Taube JM, 2014, CLIN CANCER RES, V20, P5064, DOI 10.1158/1078-0432.CCR-13-3271	2014	33.35	2015	2019	▂▂▂▂▂▂▂▂▂▂▂▂▂▂▂▃▃▃▃▃▂▂▂
16	Muzny DM, 2012, NATURE, V487, P330, DOI 10.1038/nature11252	2012	31.2	2015	2017	▂▂▂▂▂▂▂▂▂▂▂▂▂▂▂▃▃▃▂▂▂▂▂
17	Le DT, 2015, NEW ENGL J MED, V372, P2509, DOI 10.1056/NEJMoa1500596	2015	169.29	2016	2020	▂▂▂▂▂▂▂▂▂▂▂▂▂▂▂▂▃▃▃▃▃▂▂
18	Rizvi NA, 2015, SCIENCE, V348, P124, DOI 10.1126/science.aaa1348	2015	44.56	2016	2019	▂▂▂▂▂▂▂▂▂▂▂▂▂▂▂▂▃▃▃▃▂▂▂
19	Larkin J, 2015, NEW ENGL J MED, V373, P1270, DOI 10.1056/NEJMoa1504030	2015	33.68	2016	2019	▂▂▂▂▂▂▂▂▂▂▂▂▂▂▂▂▃▃▃▃▂▂▂
20	Borghaei H, 2015, NEW ENGL J MED, V373, P1627, DOI 10.1056/NEJMoa1507643	2015	32.86	2016	2020	▂▂▂▂▂▂▂▂▂▂▂▂▂▂▂▂▃▃▃▃▃▂▂
21	Brahmer J, 2015, NEW ENGL J MED, V373, P123, DOI 10.1056/NEJMoa1504627	2015	28.56	2016	2019	▂▂▂▂▂▂▂▂▂▂▂▂▂▂▂▂▃▃▃▃▂▂▂
22	Droeser RA, 2013, EUR J CANCER, V49, P2233, DOI 10.1016/j.ejca.2013.02.015	2013	27.36	2016	2018	▂▂▂▂▂▂▂▂▂▂▂▂▂▂▂▂▃▃▃▂▂▂▂
23	Motzer RJ, 2015, NEW ENGL J MED, V373, P1803, DOI 10.1056/NEJMoa1510665	2015	26.38	2016	2020	▂▂▂▂▂▂▂▂▂▂▂▂▂▂▂▂▃▃▃▃▃▂▂
24	Guinney J, 2015, NAT MED, V21, P1350, DOI 10.1038/nm.3967	2015	48.28	2017	2020	▂▂▂▂▂▂▂▂▂▂▂▂▂▂▂▂▂▃▃▃▃▂▂
25	Sivan A, 2015, SCIENCE, V350, P1084, DOI 10.1126/science.aac4255	2015	26.72	2018	2020	▂▂▂▂▂▂▂▂▂▂▂▂▂▂▂▂▂▂▃▃▃▂▂

#### Keywords and burst terms

As an important part of an article, keywords often represent its central idea and core content (31), so the analysis of keywords can help to reveal the key themes and active areas of ICB in CRC research. CiteSpace was used to generate the keyword co-occurrence network graph (**Figure 6C**) and keyword timeline graph. **Table 7** presents the top 20 keywords based on their frequency of occurrence in the 6,718 publications included in this study. Among these keywords, the most frequent ones (excluding those specified in our search strategy) were microsatellite instability (n=738), T cell (n=689), dendritic cell (n=576), survival (n=547), tumor microenvironment (n=443), breast cancer (n=397), chemotherapy (n=375), open-label (n=367), and regulatory T cell (n=365), highlighting their significance in this research area. The keyword timeline clustering graph is shown in **Figure 6D**, which can clearly shows the development of keywords in each cluster by clustering them on the timeline. This visualization not only illustrates the temporal evolution of research topics but also helps identify shifts in research focus over time. Most importantly, it facilitates us to see the period of a particular topic in a research field and helps us to explore the evolutionary trajectory of the field. In line with the co-citation clustering algorithm, here we show the top 16 clusters based on the keywords, which, after removing the clusters related to the set search strategy, are in order: #0 mismatch repair, #1 dendritic cell, #2 breast cancer, #5 cancer stem cells, #6 hepatocellular carcinoma, #7 gut microbiota, #8 receptor, #9 tumor microenvironment, #10 Incrna, #11 microsatellite instability, #12 nivolumab. These clusters collectively highlight the current trajectory and multidisciplinary nature of ICB research in CRC.

**Table 7:** Top 20 keywords.

Rank	Keywords	Records	Centrality
1	colorectal cancer	3121	0
2	expression	1173	0.04
3	immunotherapy	1120	0
4	colon cancer	1055	0.05
5	microsatellite instability	738	0.02
6	t cell	689	0
7	cell	581	0.05
8	dendritic cell	576	0.18
9	survival	547	0.11
10	tumor	522	0.13
11	therapy	515	0.21
12	tumor microenvironment	443	0.12
13	cancer	402	0.31
14	carcinoma	399	0.1
15	breast cancer	397	0.09
16	blockade	377	0.06
17	chemotherapy	375	0.13
18	open label	367	0.03
19	regulatory t cell	365	0.05
20	pd 1 blockade	354	0.01

**Figure 7:**
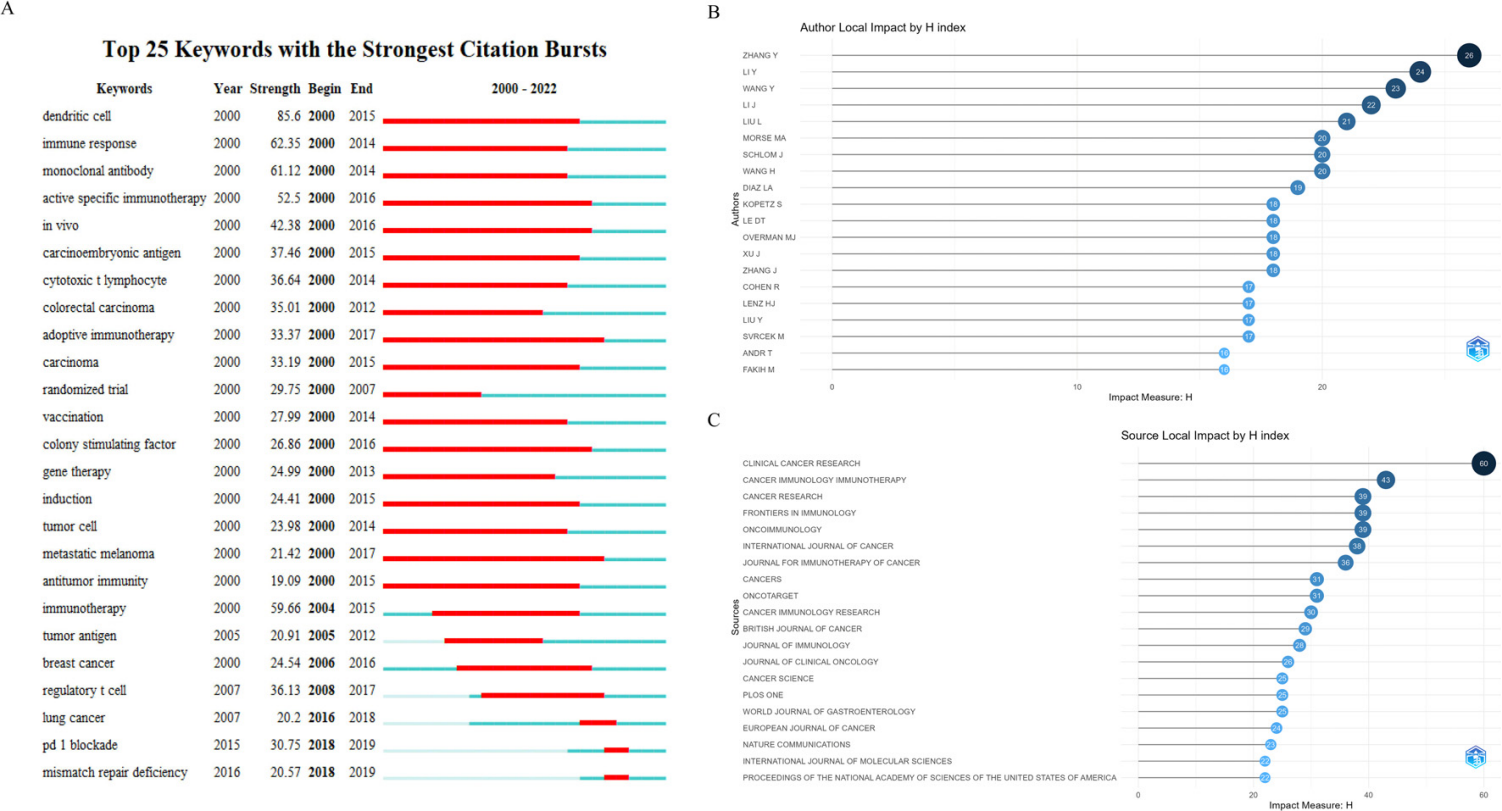
**(A)** Top 25 keywords with the strongest citation bursts. **(B)** Top 20 H-index authors. **(C)** Top 20 H-index journals.

Burst words are those that experience a rapid surge in usage within a short timeframe. Analyzing burst words provides insights into the popularity trends and temporal distribution of keywords, aiding researchers in predicting research frontiers. In **Figure 7A**, we present the top 25 burst words with the highest citation intensity among the 6,718 publications, organized chronologically. Terms like adoptive immunotherapy, colony-stimulating factor, metastatic melanoma, etc., were prominent research hotspots before 2019. By 2019, however, PD-1 blockade and mismatch repair deficiency emerged as new hotspots, indicating a shift towards immunotherapy strategies in CRC research.

#### Top 20 H-index authors and journals

The H-index, proposed by American physicist Jorge E. Hirsch in 2005, is a metric for assessing scholarly achievement and impact (32). It aims to comprehensively evaluate the quantity of publications and their citation counts, reflecting the academic influence and quality of scholarly output (33). In essence, if an author or journal has N publications, among which h papers have been cited at least h times while the remaining (N-h) papers have not been cited more than h times, then the H-index is h.

In this study, as shown in **Figures 7B, C**, we employed R-bibliometrix to identify the top 20 scholars and journals ranked by the H-index in the domain of ICB research in CRC. ZHANG Y holds the top position with an H-index of 26, followed by LI Y (H-index=24) and WANG Y (H-index=23), highlighting the significant impact of their published articles in this field.

Regarding journal H-indices, *“Clinical Cancer Research”* holds the top position (H-index=60), followed closely by *“Cancer Immunology, Immunotherapy”* (H-index=43) and *“Cancer Research”* (H-index=39). It is noteworthy that these highly-ranked journals in terms of H-indices also boast high impact factors, suggesting that the articles they publish may receive a significant number of citations in this field.

## Discussion

### General information

As the third most common malignant tumor in the digestive tract globally, 40% of CRC cases are diagnosed at an early stage (34). While surgery remains the cornerstone of treatment for this disease, patients with disease recurrence and metastasis should undergo chemotherapy. Despite advances in treatment methods including targeted therapy, the prognosis for advanced CRC remains unfavorable (35). Increasing evidence suggests that blocking immune checkpoint molecules and activating anti-tumor immune responses is one of the most promising therapeutic approaches (36, 37).

In this era of information explosion, maintaining industry leadership and staying abreast of the latest research findings has become increasingly challenging (18). To showcase the current global scientific achievements in the field of ICB in CRC, we conducted a comprehensive search of literature related to this topic published in the WoSCC from the year 2000 to 2022. Ultimately, a comprehensive bibliometric analysis was conducted on 6,718 publications from 639 countries/regions. To the best of our knowledge, this is the first bibliometric study conducted specifically on ICB in the CRC field.

Over the past 20 years, we have observed a significant increase in the number of publications and citations related to ICB therapy in the field of CRC research. Post-2019, there has been an exponential surge in publications globally regarding ICB in CRC research. Our bibliometric analysis highlights this trend, underscoring the growing interest and evolving understanding in this research domain. Therefore, it is reasonable to assume that this field will enter a golden age in the coming years.

ICB therapies, such as the well-known PD-1 and PD-L1 therapies, as well as CTLA-4 therapy, have shown promising efficacy in improving CRC. However, not all CRC patients are suitable candidates for ICB treatment. Our analysis provides insights into the current status and potential future research hotspots of ICB therapy in the CRC field, aiding in identifying the current limitations in this area and offering a more comprehensive and objective academic perspective for researchers and investors.

The research findings indicate that China leads in terms of publication volume, while the USA is ahead in total citation counts and has the most productive authors, suggesting the critical roles played by both countries in advancing ICB therapy in CRC research. Additionally, countries such as Japan, Italy, Germany, and the UK have also made substantial contributions to the development of this field. It is noteworthy that among the top 10 institutions in terms of publication volume, 5 are from China, with the remaining 5 occupied by USA-based research institutions. However, the citation counts of articles from China are significantly lower than those from the USA. Equally important, the Chinese government has acknowledged this issue and has implemented several measures to improve the quality of academic publications. Regarding national/regional cooperation networks, the USA has established partnerships with numerous countries/regions globally, highlighting the extensive global impact of ICB therapy in CRC research.

Professor Thierry André from Sorbonne University in France is the most prolific author in terms of publications and holds key leadership roles in this field, focusing primarily on gastrointestinal malignancies. Additionally, he is a primary investigator in several phase I, II, and III clinical trials evaluating therapeutic agents for gastrointestinal malignancies. Professor Heinz-Josef Lenz from the University of Southern California Norris Comprehensive Cancer Center ranks second. Professor Lenz has been dedicated to developing innovative drugs for gastrointestinal malignancies in preclinical models and initiated the first prospective randomized phase II trial using FFPE specimen gene expression results. Moreover, he discovered that the primary tumor site in CRC is an independent prognostic indicator, now included in the National Comprehensive Cancer Network (NCCN) Clinical Practice Guidelines (NCCN guidelines).

Romain Cohen ranks third in terms of publication volume, also from Sorbonne University like Professor André. He is a core member of GERCOR (Groupe Coopérateur Multidisciplinaire en Oncologie), and interestingly, Professor André is the leader of this cooperative group. Professor Cohen’s research focuses on BRAFV600E mutation in metastatic CRC patients and the impact of microsatellite instability on stage III colon cancer patients. Regarding author collaboration networks, Professor André has established stable collaborations with several prolific authors, which contributes significantly to his high academic impact and underscores his important role in advancing this research field. His team’s articles are more likely to be published in top-tier journals.

In terms of journals, **Table 4** lists the top 10 journals with the highest number of publications on this topic, as well as the top 10 journals with the highest total citations in this research field, which may represent the major journals for ICB research in CRC. Journals with high total citations generally have high quality, with 8 out of 10 having an IF higher than 10, including The New England Journal of Medicine (IF2023, 96.2), Nature Medicine (IF2023, 58.7), Nature (IF2023, 50.5), Science (IF2023, 44.7), Journal of Clinical Oncology (IF2023, 42.1), Clinical Cancer Research (IF2023, 10), Cancer Research (IF2023, 12.5), and PNAS (IF2023, 9.4). At the same time, we observed a positive co-citation relationship among both the top 10 journals in terms of publication volume and the top 10 journals in terms of total citations.

This co-citation relationship may reflect the interconnectedness and shared foundations of research within this field. Journals with higher total citations not only attract high-quality research but also become central hubs in the academic network, facilitating the dissemination and cross-referencing of key findings. The high impact factors of these journals suggest that they are platforms for cutting-edge research, which is often the most frequently cited due to its influence on subsequent studies. Moreover, these journals, particularly those like The New England Journal of Medicine, Nature Medicine, and Journal of Clinical Oncology, often publish studies with substantial clinical implications and translational potential, making them highly relevant to researchers and clinicians alike.

### Knowledge base

Co-cited literature refers to documents that are commonly cited together by multiple publications in a particular field. These articles typically address the characteristics, biological properties, classification, function, and important research mechanisms related to the study subject, often indicating groundbreaking or summarizing significance within the field (38). These co-cited papers form a “knowledge base” that underpins advancements in research. **Table 5** lists the top 10 most cited papers, and the knowledge background and reported research results covered in these papers may hold significant importance for research on immune ICB in the field of CRC.

One of the foundational studies in this area is the work of Dung T. Le et al. (22), which evaluated the efficacy of PD-1 blockade in cancers with MMR. Their findings not only highlighted a subgroup of CRC that responds favorably to ICB but also pointed toward the integration of MMR testing as a potential biomarker for patient stratification. This connection between cancer genetics and immunotherapy established a crucial pathway for identifying patients most likely to benefit from these treatments, offering a targeted solution to the variability in response rates across different tumor types.

Michael J. Overman and colleagues advanced this understanding by showing that the combination of Nivolumab and Ipilimumab yielded superior outcomes in patients with dMMR/MSI-H metastatic CRC compared to monotherapy (23). Their work underscores the importance of combination therapies in enhancing efficacy and optimizing patient outcomes, particularly in challenging cases such as metastatic disease.

Another crucial element in CRC immunotherapy is the tumor immune microenvironment. Research by Nicolas J. Llosa et al. (25). provided key insights by linking MMR status with specific immune characteristics, such as elevated Th1/CTL immune activity. This association revealed why MSI tumors, despite having an active immune microenvironment, are not naturally cleared, suggesting that targeted immune checkpoint inhibition may offer selective benefits for these subtypes of CRC. Such findings lay the groundwork for personalizing treatment strategies based on the tumor’s immune and genetic profile.

Although PD-1 inhibitors have shown clinical benefits in MSI-H or dMMR tumors after treatment, the efficacy of PD-1 inhibitors compared to chemotherapy as first-line treatment for patients with MSI-H-dMMR advanced or metastatic CRC remains unclear. Therefore, Professor Thierry André and colleagues conducted a randomized, open-label phase 3 clinical trial, including a total of 307 treatment-naive MSI-H-dMMR metastatic CRC patients. After a 2-year follow-up, the median overall survival was 13.7 months in the pembrolizumab group and 10.8 months in the chemotherapy group. There were 56 deaths in the pembrolizumab group and 69 deaths in the chemotherapy group. The overall response rate was 43.8% in the pembrolizumab group and 33.1% in the chemotherapy group. Within the overall response rate, 83% of patients in the pembrolizumab group experienced ongoing remission compared to 35% in the chemotherapy group. Grade 3 or higher treatment-related adverse events occurred in 22% of the pembrolizumab group and 66% of the chemotherapy group. This pivotal trial not only provides clear evidence for the use of pembrolizumab in specific CRC populations but also highlights the potential to shift treatment paradigms away from traditional chemotherapy for these patients.

The role of the immune system in cancer prognosis has been acknowledged for over a century, but only in recent years has it started to influence clinical decision-making. The International Immuno-Oncology Society’s evaluation of the Immunoscore in CRC offers a refined tool for assessing recurrence risk based on immune infiltration, which could complement traditional TNM staging systems. This approach points to the need for integrating immune parameters into cancer classification systems, offering a more nuanced approach to predicting patient outcomes (28).

One of the remaining obstacles in ICB treatment is primary resistance (14). The groundbreaking study by Bertrand Routy et al. identified the gut microbiota as a key factor influencing the efficacy of PD-1 inhibitors. This discovery opens new avenues for enhancing ICB therapy by modulating the microbiome, offering potential solutions for patients who do not initially respond to treatment.

In summary, these highly cited studies form the core of CRC immunotherapy research, addressing both foundational concepts and emerging challenges. Their clinical significance is underscored by the fact that the majority are clinical trials, emphasizing the ongoing need to translate scientific discovery into practical solutions. By highlighting these pivotal works, our study seeks to provide researchers and clinicians with a deeper understanding of the evolving landscape of ICB in CRC and potential strategies for overcoming current limitations in treatment.

### Emerging topics

Keywords are emphasized and highlighted in an article to capture its core content. By analyzing these keywords, we can derive the evolving focus of research topics within this field (15). We analyzed keywords and burst terms. The results indicate significant thematic changes in the ICB’s role in CRC research over the past 20 years. The main trends are manifested in the following two aspects:

### From basic research to clinical application

Our analysis highlights a clear progression in the development of ICB therapies, from early mechanistic studies to clinical applications. Initial research focused heavily on the molecular mechanisms underlying ICB, particularly with CTLA-4, and expanded to include *in vivo* experiments that evaluated the efficacy of new drugs. The rise in burst terms related to clinical trials reflects a significant transition toward testing ICB therapies in human subjects. The emergence of terms related to adverse events, such as immune-related adverse events (irAEs), underscores the growing attention to the safety and side effects associated with immunotherapy. This shift suggests that as ICB theories mature, the research focus has moved beyond basic efficacy studies to practical challenges in clinical implementation, such as optimizing treatment outcomes and minimizing adverse effects.

At a deeper level, this trend reveals the bottlenecks in the clinical application of ICB therapy. Although ICB has shown efficacy in advanced CRC patients, its effectiveness remains constrained by issues related to adverse reactions and the sustainability of therapeutic outcomes (39). Moreover, the integration of personalized medicine could help mitigate some of these challenges by identifying patients at higher risk for irAEs and adjusting treatment protocols accordingly. This proactive approach allows for better monitoring and management of potential side effects, enhancing the overall safety and effectiveness of treatment. Therefore, future research should not only focus on optimizing therapeutic efficacy but also emphasize a deeper understanding of the mechanisms underlying irAEs and how to predict and manage these reactions to achieve safer and more effective treatment.

In addition, this trend emphasizes the increasing importance of personalized medicine in tailoring ICB therapies to specific CRC subtypes. By leveraging genetic profiling and biomarker identification, researchers can better understand which patients are likely to respond positively to ICB treatment. For instance, the identification of specific mutations or immune signatures can guide clinicians in selecting appropriate candidates for ICB therapy, thereby enhancing treatment efficacy. Personalized medicine can also facilitate the development of stratified treatment plans, allowing healthcare providers to adjust ICB protocols based on individual tumor characteristics, such as immune microenvironment and tumor burden. Furthermore, it can improve treatment outcomes by enabling dose adjustments and combination therapies tailored to patients’ immune responses.

### From generalized treatment to precision medicine

Another significant theme revealed by keyword analysis is the shift from broad-spectrum ICB treatments to precision medicine approaches. In the early stages, research primarily explored the general application of ICB in CRC. However, more recent studies have focused on stratifying patients based on genetic and molecular characteristics, particularly differentiating between mismatch repair-deficient (dMMR) and microsatellite instability-high (MSI-H) tumors. The advent of precision medicine has led to the identification of distinct CRC subtypes that respond differently to ICB therapies. Keyword trends indicate that the field has progressed from identifying these subtypes to designing targeted therapies and conducting clinical trials to validate their effectiveness. As the field continues to evolve, the integration of ICB therapy with genetic and molecular profiling represents a key advancement in personalized cancer treatment (40).

While ICB therapy demonstrates promising therapeutic effects in dMMR/MSI-H mCRC, its efficacy in other subtypes remains a topic of ongoing investigation (41). Particularly noteworthy is the fact that the majority of CRC patients fall into the pMMR or MSS subtype, suggesting potential limitations in the effectiveness of ICB therapy for this population (42). Consequently, researchers have redirected their focus toward understanding immune escape mechanisms in pMMR/MSS CRC. Further in-depth clinical research into the combined use of ICB and other treatment modalities may provide valuable therapeutic insights for patients with pMMR/non-MSI-H CRC (43).

### Current research dilemmas and future directions

Although ICB has demonstrated substantial potential in the treatment of CRC, keyword analysis has also highlighted several unresolved challenges. Firstly, the efficacy of ICB therapy remains largely confined to specific subtypes, such as dMMR/MSI-H, while the majority of CRC patients fall into the pMMR/MSS subtype (44). This necessitates a deeper investigation into the limitations of ICB therapy in this patient population and the mechanisms of immune escape. Secondly, the high incidence of irAEs further restricts the broad application of ICB therapy, emphasizing the need for research focused on balancing efficacy with safety.

In light of these challenges, future research should advance in several key areas: First, the identification of additional subtypes of patients who could benefit from ICB therapy through new molecular markers and bioinformatics technologies; second, the development of combination strategies with ICB, such as combined chemotherapy, targeted therapy, or novel immunomodulators, to address the efficacy limitations of ICB in pMMR/MSS CRC patients; and finally, enhancing the prediction, monitoring, and management of irAEs to minimize the occurrence of adverse reactions. These directions not only have the potential to advance CRC immunotherapy but also offer insights for the treatment of other types of cancers.

### Strengths and limitations

Clinical physicians have long prioritized enhancing clinical therapies for CRC and improving patient prognosis (45). With advances in tumor cell biology research, ICB has garnered significant attention in the treatment of malignant tumors, yielding notable efficacy in certain CRC subtypes. Despite numerous publications on ICB in the CRC domain, there has been no literature-based bibliometric analysis conducted in this area. This study delves into an in-depth analysis of the research direction and prospects of ICB in the CRC domain; however, it also has limitations. Firstly, the literature search was limited to the core dataset of the WOS, focusing solely on English-language publications, which may inherently bias our findings and potentially exclude relevant original works published in other languages. This bias can lead to an incomplete understanding of the global research landscape, particularly in regions where non-English publications are prevalent. Furthermore, the bibliometric analysis method employed is only applicable to general information and does not encompass full-text content, potentially resulting in the omission of crucial details such as author viewpoints and future perspectives. Additionally, our analysis predominantly featured highly cited papers, which, while reflecting significant contributions to the field, may overlook important studies that have not yet received widespread recognition. To address these limitations, future research endeavors will expand the scope of data collection, including non-English publications and lesser-cited studies, enhance discoverability, and provide more valuable insights and support for researchers.

## Conclusion

In recent years, immunotherapy has made remarkable progress, achieving significant advancements in the treatment of CRC, whether in MSI-H/dMMR or pMMR/non-MSI-H subtypes and regardless of whether it is used in neoadjuvant therapy for locally advanced disease or palliative therapy for late-stage disease. Immunotherapy, represented by ICB, has shown notable progress in the CRC domain, underscoring its significant therapeutic potential and research value.

In this study, employing bibliometric tools, we objectively delineate for the first time the research trends in this domain, including the annual publication growth trends, co-occurrence networks of countries and institutions, authors and co-cited authors, keywords, and co-cited patterns of highly cited literature. Through comprehensive bibliometric analysis, we ascertain the current status of research in this domain and identify key research themes and hotspots for the future.

Currently, research on ICB in CRC primarily focuses on exploring and formulating corresponding immunotherapy (combination) regimens based on different subtypes of CRC. It is noteworthy that MSI is currently the only approved biomarker for screening CRC immunotherapy, while other immunotherapy biomarkers have limitations that require substantial clinical research for validation. Additionally, optimizing biomarker detection methods and establishing uniform assessment criteria are needed to facilitate the stratification of CRC patients, improve patient prognosis and overall survival rates, and promote the development of precise biomarkers and precision medicine. Finally, the prediction, monitoring and management of irAEs should be enhanced to minimise the occurrence of adverse reactions. Overall, the findings of this study are expected to provide valuable insights for expert decision-making and funding support.

## Data Availability

The original contributions presented in the study are included in the article/**Supplementary Material**. Further inquiries can be directed to the corresponding author/s.
