# Supplementary material for: Insights into the historical trajectory and research trends of immune checkpoint blockade in colorectal cancer: visualization and bibliometric analysis
Source: Front Immunol. 2024 Oct 31;15:1478773. doi: 10.3389/fimmu.2024.1478773 (PMC11560439; doi:10.3389/fimmu.2024.1478773)
Supplement: Supplementary file 1 [file Table1.docx]

Immune checkpoint blockade search term:

| 1 | TS=(ipilimumab) OR TI=(ipilimumab) OR AB=(ipilimumab) |
| --- | --- |
| 2 | TS=(pembrolizumab) OR TI=(pembrolizumab) OR AB=(pembrolizumab) |
| 3 | TS=(nivolumab) OR TI=(nivolumab) OR AB=(nivolumab) |
| 4 | TS=(immunotherapy) OR TI=(immunotherapy) OR AB=(immunotherapy) OR TS=(immune checkpoint blockade) OR TI=(immune checkpoint blockade) OR AB=(immune checkpoint blockade) OR TS=(immune checkpoint inhibitor) OR TI=(immune checkpoint inhibitor) OR AB=(immune checkpoint inhibitor) |
| 5 | TS=(PD-1) OR TI=(PD-1) OR AB=(PD-1) OR TS=(PD-L1) OR TI=(PD-L1) OR AB=(PD-L1) OR TS=(CTLA-4) OR TI=(CTLA-4) OR AB=(CTLA-4) |
| 6 | TI=(yervoy) OR AB=(yervoy) OR TI=(keytruda) OR AB=(keytruda) OR TI=(opdivo) OR AB=(opdivo) |
| 7 | #1 OR #2 OR #3 OR #4 OR #5 OR #6 |

Colorectal cancer search term:

| 8 | TS=("Rectal Neoplasm" OR "Rectal Tumor" OR "Rectal Cancer" OR "Rectum Neoplasm" OR "Rectum Cancer" OR "Cancer of the Rectum" OR "Cancer of Rectum" OR "Colorectal Neoplasm" OR "Colorectal Tumor" OR "Colorectal Cancer" OR "Colorectal Carcinoma" OR "Colonic Neoplasm" OR "Colon Neoplasm" OR " Cancer of Colon" OR "Colon Cancer" OR "Cancer of the Colon" OR "Colonic Cancer" OR "CRC") OR TI=("Rectal Neoplasm" OR "Rectal Tumor" OR "Rectal Cancer" OR "Rectum Neoplasm" OR "Rectum Cancer" OR "Cancer of the Rectum" OR "Cancer of Rectum" OR "Colorectal Neoplasm" OR "Colorectal Tumor" OR "Colorectal Cancer" OR "Colorectal Carcinoma" OR "Colonic Neoplasm" OR "Colon Neoplasm" OR " Cancer of Colon" OR "Colon Cancer" OR "Cancer of the Colon" OR "Colonic Cancer" OR "CRC") OR AB=("Rectal Neoplasm" OR "Rectal Tumor" OR "Rectal Cancer" OR "Rectum Neoplasm" OR "Rectum Cancer" OR "Cancer of the Rectum" OR "Cancer of Rectum" OR "Colorectal Neoplasm" OR "Colorectal Tumor" OR "Colorectal Cancer" OR "Colorectal Carcinoma" OR "Colonic Neoplasm" OR "Colon Neoplasm" OR " Cancer of Colon" OR "Colon Cancer" OR "Cancer of the Colon" OR "Colonic Cancer" OR "CRC") |
| --- | --- |

#7 AND #8

Time: 2000-01-01-2023-01-01.

Database: WOS core.

Language = English

(TS=(ipilimumab OR pembrolizumab OR nivolumab OR immunotherapy OR “immune checkpoint blockade” OR “immune checkpoint inhibitor” OR PD-1 OR PD-L1 OR CTLA-4) OR TI=(ipilimumab OR pembrolizumab OR nivolumab OR immunotherapy OR “immune checkpoint blockade” OR “immune checkpoint inhibitor” OR PD-1 OR PD-L1 OR CTLA-4 OR yervoy OR Keytruda OR opdivo) OR AB=(ipilimumab OR pembrolizumab OR nivolumab OR immunotherapy OR “immune checkpoint blockade” OR “immune checkpoint inhibitor” OR PD-1 OR PD-L1 OR CTLA-4 OR yervoy OR Keytruda OR opdivo) ) AND (TS=("Rectal Neoplasm" OR "Rectal Tumor" OR "Rectal Cancer" OR "Rectum Neoplasm" OR "Rectum Cancer" OR "Cancer of the Rectum" OR "Cancer of Rectum" OR "Colorectal Neoplasm" OR "Colorectal Tumor" OR "Colorectal Cancer" OR "Colorectal Carcinoma" OR "Colonic Neoplasm" OR "Colon Neoplasm" OR " Cancer of Colon" OR "Colon Cancer" OR "Cancer of the Colon" OR "Colonic Cancer" OR "CRC") OR TI=("Rectal Neoplasm" OR "Rectal Tumor" OR "Rectal Cancer" OR "Rectum Neoplasm" OR "Rectum Cancer" OR "Cancer of the Rectum" OR "Cancer of Rectum" OR "Colorectal Neoplasm" OR "Colorectal Tumor" OR "Colorectal Cancer" OR "Colorectal Carcinoma" OR "Colonic Neoplasm" OR "Colon Neoplasm" OR " Cancer of Colon" OR "Colon Cancer" OR "Cancer of the Colon" OR "Colonic Cancer" OR "CRC") OR AB=("Rectal Neoplasm" OR "Rectal Tumor" OR "Rectal Cancer" OR "Rectum Neoplasm" OR "Rectum Cancer" OR "Cancer of the Rectum" OR "Cancer of Rectum" OR "Colorectal Neoplasm" OR "Colorectal Tumor" OR "Colorectal Cancer" OR "Colorectal Carcinoma" OR "Colonic Neoplasm" OR "Colon Neoplasm" OR " Cancer of Colon" OR "Colon Cancer" OR "Cancer of the Colon" OR "Colonic Cancer" OR "CRC"))
